# Exploring factors that affect the uptake and sustainability of videoconferencing for healthcare provision for older adults in care homes: a realist evaluation

**DOI:** 10.1186/s12911-020-01372-y

**Published:** 2021-01-06

**Authors:** Louise Newbould, Steven Ariss, Gail Mountain, Mark S. Hawley

**Affiliations:** 1 Social Policy Research Unit (SPRU), University of York, Heslington, York YO10 5DD UK; 2 Centre for Assistive Technology and Connected Healthcare and School of Health and Related Research, The University of Sheffield, Regent Court, 30 Regent Street, Sheffield, S1 4DA UK; 3 Centre for Applied Dementia Studies, University of Bradford, Bradford, UK

**Keywords:** Videoconferencing, Care homes, Older adults, Admissions, Implementation, Sustainability, Uptake, Remote consultation

## Abstract

**Background:**

Videoconferencing has been proposed as a way of improving access to healthcare for older adults in care homes. Despite this, effective uptake of videoconferencing remains varied. This study evaluates a videoconferencing service for care home staff seeking support from healthcare professionals for the care of residents. The aim was to explore factors affecting the uptake and sustainability of videoconferencing in care homes, to establish what works for whom, in which circumstances and respects. The findings informed recommendations for commissioners and strategic managers on how best to implement videoconferencing for remote healthcare provision in care homes for older adults.

**Methods:**

Realist evaluation was used to develop, refine and test theories around the uptake and maintenance of videoconferencing in three care homes across Yorkshire and the Humber, England. The care homes were selected using maximum variation sampling regarding the extent to which they used videoconferencing. A developmental inquiry framework and realist interviews were used to identify Context, Mechanism and Outcome Configurations (CMOCs) regarding uptake and sustainability of the service. Participants included care home residents (aged > 65) and staff, relatives and strategic managers of care home chains. The interviews were an iterative process conducted alongside data analysis. Transcripts of audio recordings were entered into NVIVO 12, initially coded into themes, then hypotheses developed, refined and tested.

**Results:**

Outcomes were generated in relation to two main contextual factors, these were: (1) communication culture in the home and (2) the prior knowledge and experience that staff have of videoconferencing. The key facilitators identified were aspects of leadership, social links within the home and psychological safety which promoted shared learning and confidence in using the technology.

**Conclusions:**

Videoconferencing is a valuable tool, but successful implementation and sustainability are dependent on care home culture and staff training to promote confidence through positive and supported experiences.

## Contributions to literature

- This paper explores key aspects of the care home context that influence the uptake and sustainability of videoconferencing for remote healthcare provision.
- It provides a valuable insight for commissioners in how best to implement the service. This will help inform managers when considering how to improve the readiness of care homes prior to implementation.
- It provides learning for service providers when considering the development/diffusion of similar services in terms of which elements of the intervention are important to improve uptake/ sustainability.

## Background

### Rationale for evaluation

The number of older adults in the UK is increasing [1], resulting in rising demand in the care home sector [2]. This grow in demand, along with funding cuts makes delivering healthcare to care home residents challenging [3]. One way of addressing this problem may be through videoconferencing (a live two-way audio-visual link between the healthcare provider and care home) [4].

The 'Five-Year Forward View’ (2014) for the National Health Service (NHS), emphasised the importance of providing new care models that are adaptable to the needs of the population, whilst harnessing the benefits of new technology in order to reduce variation in the quality of healthcare [5]. In the NHS Long-term Plan (2019), the importance of expanding support for older adults living in care homes and better utilising technology to provide more convenient access to care was reiterated [6].

Videoconferencing has been shown to remove geographical barriers to care [7], whilst improving continuity of care [8] and access for those with physical disabilities [9]. It has been shown to improve access to a range of services [8]; allowing the assessment of residents before a possible admission to hospital [4]. It can also enhance access to primary care [10, 11]; it can be adapted to the health needs of the population; and allows support to be delivered 24 h a day, seven days a week, from health professionals at a remote site [9]. Suggested benefits also include improved staff confidence [12] and the opportunity for older adults to achieve a ‘good death' in the surroundings of their own home and family [13], as described by the End of Life Care Strategy [14]. An evaluation of telehealth in care homes indicated promise for reducing bed stays in hospital, admissions, and emergency department attendances for older people [4].

However, some research has also found there to be limited potential in certain aspects [4, 15, 16]. For example, an evaluation report by Hex and Wright (2015) found that although videoconferencing reduced hospital admissions from care homes, the data also suggested an increase in mortality associated with non-elective admissions. However, these findings were not conclusive; there is a question over attribution and whether other factors may have been responsible for the observed outcomes [4]. Findings from Flodgren (2015) showed inconsistencies in the effectiveness of videoconferencing in different patient groups when compared to face-to-face services [16].

Whilst videoconferencing has demonstrated feasibility in care home settings [17], research into the use of videoconferencing in the UK is scarce, with the majority being small-scale case studies (> 50%) [18]. The uptake of technological interventions in care homes has varied greatly [19]. This has been attributed to a range of factors, such as leadership, culture, organisational slack (staff, space and time) and social capital (active connections between staff) [20].

### Evaluation questions, objectives and focus

The aim of this research was to explore the factors affecting the uptake and sustainability of videoconferencing in care homes, to establish what works for whom, in which circumstances and respects.

## Methods

### Programme theory

In order to evaluate videoconferencing, the initial programme theories for the intervention were identified first, to inform the further development and testing of hypotheses [21].

Key texts were identified to inform some initial programme theory [18] and were explored for their potential to inform theory development. However, the majority of literature focused on intervention characteristics (e.g. the technology) and was therefore deemed unsuitable.

The middle range theories below were derived from initial theory gleaning interviews undertaken with staff in care home one [22]. Emerging concepts were initially identified using a grounded theory approach [23, 24] to describe key themes and mapped into further subthemes, these were broadly:

1. level of expertise amongst staff (nursing staff working at the home (no.) years practicing in the UK, familiarity of resident needs, access to available services),
2. health of the residents (ability to travel, likelihood of presenting with acute symptoms)
3. managerial support (awareness/knowledge of videoconferencing, support after negative experiences using the technology, guidance over when to use the service over other available services)
4. technical equipment (communication—view of resident & perceived presence of the professional).

These themes were then explored to construct initial programme theories for further development, testing and refinement throughout [21, 24].

### Rationale for using realist evaluation

Due to a wide range of variables that could influence the uptake and sustainability of videoconferencing (the key outcomes in our theory development), realist evaluation, as described by Pawson (1997) was used to accommodate the complex nature of the setting [25]. It should also be recognised that it is a social intervention that relies on stakeholder engagement to produce wide-ranging outcome patterns [21]. Therefore, the intervention, in addition to the setting, is complex. Pawson (2013) outlines seven key considerations for complex evaluations, which provided a framework to incorporate the complexities of the settings and interventions. These are volitions, implementation, contexts, time, outcomes, rivalry, and emergence. A realist approach is particularly suited to address these elements of complexity. It helps identify why interventions do and do not work for certain people in certain contexts, and how different outcome patterns are produced [21].

An 'inquiry framework' proposed by Patton (2011), was used in individual interviews to build up a more comprehensive picture of videoconferencing in care homes, it asked ‘who, what, where, when, how, and by what means?’ [26].

### Surroundings

This project purposefully selected three care homes in Yorkshire and the Humber. Care homes are comprised of residential/nursing homes that provide care and support day and night, with nursing homes offering additional 24-h care from nursing staff for residents who are more dependent [27].

### Programme being evaluated

Associated with the Five-Year Forward View report [5], for enhanced care in care homes, NHS England funded the implementation of six New Care Model Vanguards. All those recruited to this study were implementing interventions through the intermediate care hub provided by vanguard model 1 [5]:

Vanguard Model 1: during the study, this service was working with approximately 44 Clinical Commissioning Groups (CCGs) and providing videoconferencing to approximately 546 care homes [28]. This model aims to improve access to healthcare and reduce unnecessary travel and hospital admissions. The intermediate ‘Care Hub' can be accessed on behalf of residents 24 h a day, seven days a week. It can be used to seek advice for any health concern, and where necessary can escalate the call to a doctor in hospital, send an ambulance or out-of-hours general medical practitioner (GP) [10, 11].

### Description and justification of the programme design

A case study design was chosen to compare and contrast the different settings and contexts. A large amount of qualitative data were collected to establish outcome patterns and test and refine theory [29, 30]. Two key strategic managers from two large care providers were consulted in order to define the boundaries of the study before data collection began [31].

**Fig. 1:**
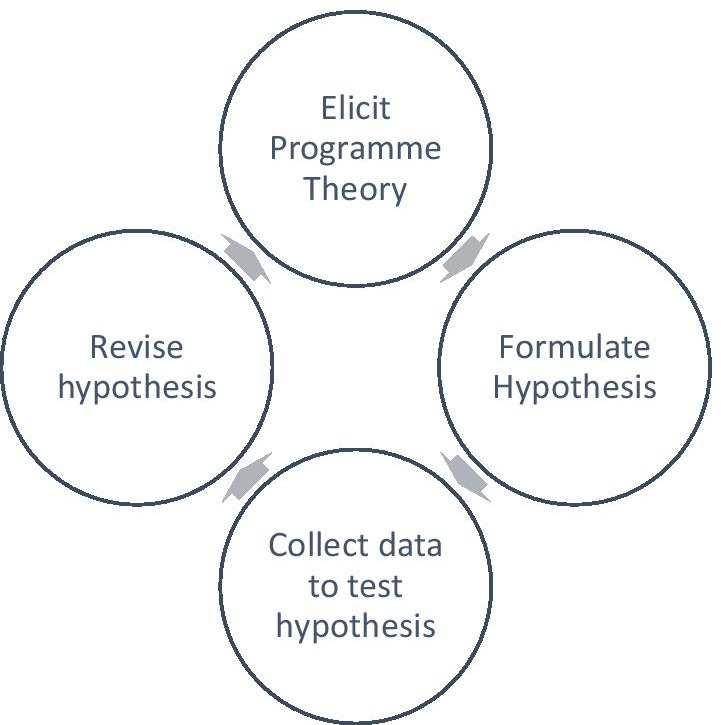
Realist Wheel of Evaluation by Pawson [21] (pp. 88)

Figure 1 shows the iterative realist evaluation cycle developed by Pawson (2013) [21].

## Data collection

### Interviews

Theory-gleaning interviews were used with a developmental inquiry framework to explore how videoconferencing produced different outcomes in each care home [26]. These were used to further develop and refine the interview guide [22]. Realist interview techniques were then used to develop, refine and test emerging hypotheses at different stages [22].

### Recruitment process and sampling strategy

Survey respondents were selected using maximum variation sampling, from a pool of participants already identified whilst undertaking a care home survey exploring attitudes to telemedicine which has been published elsewhere [32].

Three care homes were selected for further study based on the length of time the care home had been using videoconferencing, the range of purposes and conditions it was being used for and the frequency of use [33]; which were identified through the survey responses.

### Analysis

Initially, the interview data were analysed thematically. In care home one, the principles of grounded theory were employed to identify key relationships, concepts, and areas for theory development [23]. Using NViVO qualitative data analysis software, free nodes were grouped into possible relationships. Areas for theory development evolved using NViVO mind maps.

For care home two, the key concepts and relationships were reformulated deductively. In light of the initial findings, the Consolidated Framework for Implementation Research (CFIR) [34] was used to review emerging themes and to provide a consistent theoretical framework [35].

The key relationships and concepts were then refined, consolidated and further tested in care home three. CMOCs were identified in the data [24] and developed using the guidance provided by Dalkin [36] on how to define a mechanism, expressed as follows:

$${\text{M }}({\text{Resources}}) \, + {\text{C}} \, ({\text{Context}}) \to {\text{M}}({\text{Reasoning}}) \, = {\text{O}}\left( {{\text{Outcome}}} \right)$$

The findings of this project are reported below, following the RAMESES II reporting standards [37, 38].

## Ethics

The proposal for this study was reviewed by the School of Health and Related Research (ScHARR) research ethics committee (REC reference: 004,413) at the University of Sheffield.

## Results

Table 1 shows the care home characteristics that determined their selection into the study.

**Table 1:** Care home characteristics

Care home	Size (beds)	District	Funding	Rural/Urban Code	Type	Videoconferencing use	Service
1	34	East Yorkshire	Private	E1 (a rural village)	Nursing	Trialling with CCG for 11 months	24 h hub
2	28	West Yorkshire	Private	D1 (a rural town fringe)	Residential	Had been using it for~ 2 years	24 h hub
3	39	South Yorkshire	Charity	B1 (minor conurbation)	Residential	Not in use	None

*Care home one* This care home had been trialling videoconferencing for 11 months at the time of this fieldwork. However, this service has since been withdrawn by the CCG as the trial found that the introduction of videoconferencing at pilot sites had increased the number of hospital admissions.

*Care home two* This home had access to a nursing service which could be contacted out of hours, any day of the week. It also had weekly visits from the district nurse. There were no qualified nurses on-site. Videoconferencing was integrated into regular practice.

*Care home three* The home had no qualified nurses on-site, however a GP also visited the home every Thursday for residents that were particularly ill. Also, a nurse visited to attend to care needs such as wounds and dressings.

### Interviews

Interviews were conducted with 25 participants. Residents were only approached to take part if they were deemed by the care staff to have capacity to consent to the study. Table 2 shows the breakdown of type of participant interviewed in each case study.

**Table 2:** Interviews conducted by care home

Care home 1 (n) ^a^	Care home 2 (n)	Care home 3 (n)
Manager (1) Nurses (3) Night nurse (1) Senior care assistant (1) Care assistant (1) Resident (1)	Manager/owner(1) Deputy manager (1) Senior care assistants (4) Day care assistants (2) Night care assistant (1) Relatives (2) Resident (1)	Manager (1) Team leader (1) Activity coordinator (1) Care assistants (2) Relatives (2)

^a^Three residents were identified as having capacity to participate, one was interviewed fully and another was unable to complete the interview. The third did not consent to take part

## Main findings

### Middle range theory

Pawson (2013) suggests that an important aspect of Realist Evaluation is the development of theories that operate at a middle range [21]; collecting data about concepts that fall between universal theory and the description of day-to-day implementation of a programme [39].

These middle-range theories (MRTs) provide a level of abstraction relating empirical data to transferrable lessons. They can be bespoke theories; specific to a particular setting or intervention. Alternatively, MRTs can be framed around pre-existing, ‘formal’ theories; which are appropriate to enhance analyses and the usability of findings. This study explored the explanatory power of a number of formal MRTs.

The first MRT identified was ‘organisational readiness for change’ (Weiner, 2009), which describes the following supportive context for innovation. “A psychological state in which organisational members feel committed to implementing an organisational change and are confident in their collective abilities to do so” [40], P.6). The theory is composed of two main elements; a shared vision to implement change (change commitment) and a shared belief in the ability to affect the change (change efficacy) [40].

To explain how interdependence in the change process might affect the uptake and sustainability of videoconferencing, individual-level theories were identified to explain the variations in implementation in different contexts.

**Fig. 2:**
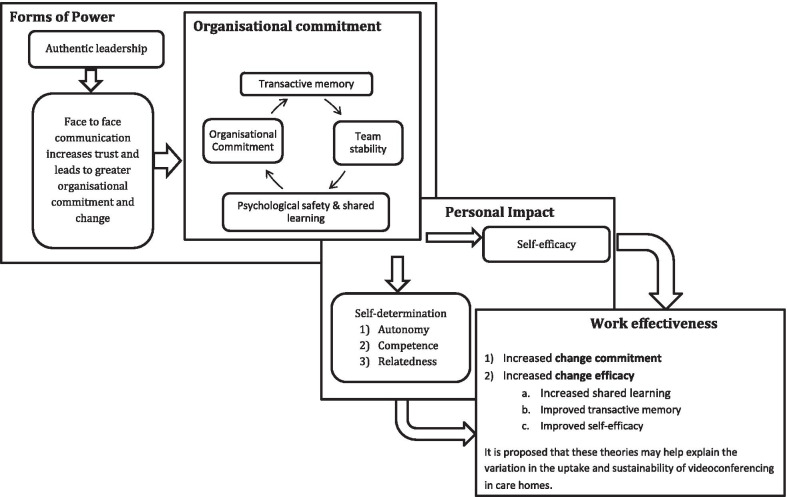
Theoretical model explored for theory development and refinement adapted from Laschinger et al. [42] (pp. 2)

MRTs that were considered to explain the emerging findings included ‘self-determination theory’ [41] and Rosabeth Kanter's (1979) ‘structural theory of empowerment’[42, 43]. Rosabeth Kanter's model is composed of three parts: forms of power, personal impact, and improved work effectiveness [42]. Figure 2 shows how individual-level theories may further explain variations in implementation in different contexts within this model.

This model explored theories to explain how adoption and sustainability of videoconferencing are related to increasing or decreasing change-commitment and change-efficacy through authentic leadership, which would include advocacy for change [44].

### Contexts

The main features of context that generated outcomes, were as follows:

- *Care home culture*, including trust and shared learning, previous opportunities to use technology, prior use of the current system, and self-efficacy/experience in delivering regular care (e.g. in communicating with residents & meeting resident needs)
- *Authentic leadership* advocacy/opinion leaders
- *Staff empowerment & promotion of self-efficacy* Information, training, and social links in the home, managerial autonomy, feeling valued, identification with the organisation
- *Care home characteristics* tension for change; and structural characteristics (size of the organisation, skill set of staff).

### Mechanisms

The key mechanisms are as follows:

- Self-efficacy [45] which includes:
  - Vicarious experience, or learning through/with others (modelling behaviour)
  - Performance outcomes (attitudes derived from the accumulation of past experience of undertaking the task)
  - Verbal persuasion (encouragement from others)
  - Physiological feedback (how one feels when completing the task)
- Psychological safety in the home
- Confidence in using the system
- Perceived benefits to resident care

These key contexts and mechanisms are all represented in the proposed model. However, when prioritised for importance, the most active and influential mechanisms relate to self-efficacy, and confidence and these are closely related to the context of social links within the home to provide opportunities for shared learning and support. Tension for change (e.g. level of dissatisfaction with current services) and structural characteristics (e.g. the staff hierarchy/composition) were also key contextual factors identified as facilitating change.

### Outcomes

The principle outcome patterns were ultimately related to whether or not videoconferencing was used, to what extent, by whom and for how long.

#### CMO configurations (CMOCs)

The project identified twenty-one CMOCs that explained variation in videoconferencing outcomes.

Presented below are a sample of five configurations relating to each aspect of the setting as defined by the CFIR (Intervention, Outer setting, Inner setting, Individual characteristics and Process) [34]. These CMOCs are summarised in Table 3.

**Table 3:** Summary of main CMOCs presented in the paper

CMOC	Mechanism (resource)	Context	Mechanism (reasoning)	Outcome
Faster response supports uptake	Quick access to services	Staff observe that fastest response	Perceived benefit in speed means it’s more likely to be used again	More experience of rapid access will increase use, and becoming part of routine care
Unequal relationships hinder use	Opportunity to reduce professional isolation	Where staff consider themselves equal partners, videoconferencing may reduce professional isolation However if staff feel inferior, and the staff are qualified nurses, they may be less likely to use the service	When staff consider themselves equal partners, videoconferencing will tend to be used sooner for advice	Videoconferencing is more likely to be used when staff feel equal and feel the need to seek support. Increasing the uptake and sustainability
Team stability & support encourages psychological safety and innovation	Provides a new and innovative way of delivering healthcare	Where staff feel unsure of how to meet a resident's needs, they may seek support from a member of staff with more experience. Where there is strong team stability, members are likely to have increased psychological safety	Here, it is more likely that the staff member will seek support before using the service. As their self-efficacy grows, it will become less daunting, and staff may begin to use the system independently	Increased self-efficacy will help to overcome obstacles and reaffirm commitment to videoconferencing. Which will make videoconferencing more sustainable
Low confidence hinders uptake	Opportunity for junior staff members to consult with a remote nurse	Where staff have less experience in managing residents’ health, they may be less confident in using videoconferencing	Less experienced staff will be more likely to access the service with the support of someone that is more experienced / in a more senior post	An adequate ratio of senior to junior employees and a culture of supporting junior staff will increase the uptake and sustainability of videoconferencing
Opinion leader advocacy supports uptake	a new method of healthcare delivery	There is a pre-existing homogenous environment, which supports the exchange of opinions	Staff act as informal / formal opinion leaders, sharing their view of the system. It is more likely that the impact of advocacy amongst the team will be increased	More staff will trial the system and develop their self-efficacy. Depending on their experiences, this may increase uptake and sustainability

Data available for all CMOCs upon request

### Intervention: relative advantage

#### 'Faster response supports uptake'

- *Mechanism (resource)* Videoconferencing provides quick access to healthcare expertise.
- *Context* In contexts where staff members recognise the need for residents to access health services quickly, and they do not receive timely responses from alternatives to videoconferencing, staff members are more likely to want to use videoconferencing. However, experiences of slow responses communicated by others might create contexts with negative influences.
- *Mechanism (reasoning)* The expectation of a relatively rapid response will make it more likely that videoconferencing is used to access healthcare, advice, and services in subsequent situations. There is a feedback loop, whereby staff members are able to see the difference in speed compared to other services, which makes it more likely that they will reaffirm their commitment to using it.
- *Outcome* When staff members use videoconferencing more, become familiar with the system, and have experiences of rapid access; the perceived ease of use will increase. This will tend towards it being used more, and becoming part of routine care; so the uptake and sustainability of videoconferencing is increased.

#### Evidence

It [the hub] is a lot more convenient and it's a lot better for the residents than ringing 111, so for the same situation I've used both, and the hubs just worked. And it's been over and done with in under two hours, and I've been waiting ten for another. (Senior carer; care home two).

However, care home one found a reduced response time when using videoconferencing:

Err yes I think, I was lucky [?] during the night, because it was so quick their answer, but I know from colleagues that [omitted] and other home [omitted] I know it's happened once or twice where they may be waiting for half an hour/40 min to get a call. So, maybe after half an hour you give up and you call the GP, or you give up and call 111. So, I think the time really [?] if it's an urgent situation you want them to be there just now, you know (Night nurse; care home one).

### Outer setting

#### 'Unequal relationships hinder use'

- *Mechanism (resource)* Videoconferencing provides the opportunity to reduce professional isolation and for staff members to communicate on an equal footing with peers.
- *Context* In contexts where care home staff feel inferior to the staff at the remote sites, and have residents that are suffering greater ill health, qualified nursing staff may be less likely to call until they have made every effort to treat the resident using support within the home. However, where they consider themselves equal partners, and they have limited support at the care home, contact through videoconferencing may help to reduce professional isolation.
- *Mechanism (reasoning)* When staff members feel that they might be judged as being incompetent or inefficient, it is more likely that contact will be delayed. However, when they consider themselves equal partners, videoconferencing will tend to be used sooner for advice.
- *Outcome* If members of staff consider themselves unequal partners, it is more likely that they will have tried to resolve issues themselves and therefore will require a speedy response when they eventually use videoconferencing. Correspondingly, if the hub does not respond quickly then confidence will be lost, and uptake and sustainability of videoconferencing will be reduced. However, if videoconferencing is used with little delay, for advice (when staff consider themselves equal partners), it is more likely that uptake and sustainability will be increased.

#### Evidence

I think sometimes because you're a bit unsure as to when to ring 111 and stuff like that because we're not nurses and we're not doctors, we're care assistants and we're not medically trained, so sometimes you do sit back and think: do I get a GP? Don't I? You sometimes feel a bit of a nuisance, but then you speak with your other team leaders and think: right, we do need to ring and get some advice. So, it would be nice to have that and not feel like you're being a pain in the backside. (Team Leader; care home three).

When asked about a conflict that had arisen between the remote site and care home, a nurse responded:

I think historically they (staff at the remote site) think that the nurses in care homes are not second rate, but that they are not as experienced, but we can hold us own. If anyone says anything to us, we can answer back. And it's like my colleague [omitted] worked [omitted] years on the same surgical ward… I've been a ward sister. So, it's not that we've not got the experience.. (Nurse; Care home one).

### Inner setting

#### 'Team stability & support encourages psychological safety and innovation'

- *Mechanism (resource)* The stability of the care home team effects attitudes towards videoconferencing in providing a new and innovative way of delivering healthcare.
- *Context* In contexts where the staff members feel unsure of how to meet a resident's needs, they may be more inclined to seek out support from a member of staff with knowledge of the resident and more care experience. Where there is strong team stability and low staff turnover, members are likely to have increased psychological safety.
- *Mechanism (reasoning)* This will make it more likely that the staff member will address their uncertainty by calling a member of staff (even if they are off-duty) before using videoconferencing. As their self-efficacy grows through time and training, knowing when to use videoconferencing will become less daunting, making it more likely that staff will use it without checking first and that the perceived ease of use of the system will increase, along with their self-efficacy. Also, making it more likely that staff will be more innovative and build up their experience.
- *Outcome* A commitment to innovation will build up self-efficacy in using videoconferencing, helping to overcome obstacles and reaffirm commitment to videoconferencing. Experiencing benefits of videoconferencing in a workplace that supports innovation can result in advocacy for the system and promotion to others. This increase in uptake, commitment and support to overcome obstacles will make it more sustainable.

#### Evidence

Care home two had a low staff turnover rate, and demonstrated a greater sense of a psychological safety:

I think more with the night staff, they tend to ring the on call first, because you might not get a very experienced person who’s used the hub. The training’s there if they want it. So I think they would edge more to ring the ‘on call’. Then if it’s something that the on call will think, I’ll come in and sort it out rather than leaving it on the shoulders of the night staff (senior carer).

One member of staff at this same home further expanded the use of videoconferencing to seek advice for their colleagues health, this demonstrates that the carer felt able to innovate:

Erm the senior member of staff had a needle stick injury and the senior member of staff wanted a bit of advice … I was there at the time observing, she wanted me to assist her in using the videoconferencing … just to see if she needed any medical attention. (Care assistant).

As well as expanding its use for residents:

Originally, when we first got it, it was more just a night thing, but as we've used it more we've found that we can really use it for anything. (Senior carer).

### Characteristics of the individual

#### 'Low confidence hinders uptake'

- *Mechanism (resource)* Videoconferencing provides the opportunity for junior staff members to consult with a remote nurse.
- *Context* In contexts where staff have less experience in managing residents’ health, they may be less confident in using videoconferencing.
- *Mechanisms (reasoning)* This means that staff who are less experienced will be less likely to access videoconferencing without the support of someone that is more experienced or in a more senior post.
- *Outcome* An adequate ratio of senior to junior employees and a culture of supporting junior staff makes it more likely that a wider range of staff members will use videoconferencing. This increases the chance that more people in the home will see a positive impact and that less senior members of staff will observe this and use the system.

#### Evidence

I think that would be a good thing for team leaders, because they're more of a first point if anything goes wrong…I can't see a care assistant particularly being confident to use it (Activity coordinator; care home three).

Erm you have support there. If you're stuck, you can always ask…Support is key, because you know if you're not confident in using something it's quite terrifying, to be honest. You don't know whether you're coming or going…. (Care assistant, care home two).

### Process: opinion leaders

#### 'Opinion leader advocacy supports uptake'

- *Mechanism (resource)* Videoconferencing is a new method of healthcare delivery with varying levels of endorsement by members of staff.
- *Context* Where there is a pre-existing homogenous environment, which supports the exchange of opinions.
- *Mechanism (reasoning)* It is more likely that specific staff members will act as informal or formal opinion leaders of videoconferencing, sharing their view of the system. Therefore, the impact of advocacy amongst the team will be increased, and so commitment to overcoming barriers to implementation will be increased.
- *Outcome* This will result in more members of staff trialling the system and developing their self-efficacy depending on their experiences, which will increase uptake and sustainability.

#### Evidence

In the home with a pre-existing homogenous environment, there was evidence of advocacy amongst less senior members of staff:

…I’d say more people should use it, because it’s 24 h, it’s open 24 h and obviously you’re not having the stress and worry of having to call out of hours (Care Assistant).

In the care home where they were struggling to utilise it, it was noted:

That’s it, as I say we did have problems, but I think they have the staffing sorted now (The manager) quite likes it and we like it as well as a team of nurses thinking ‘oh yeh let’s try this’ (nurse).

Here the lack of reference to the ‘care home team’ e.g. carers, demonstrates a divide with carers being unsure how the system works:

Erm I have a rough idea, how and why they use it. Obviously the telemed, I feel it’s erm. …like a conferencing between themselves here or a doctor and it would be a doctor or a nurse elsewhere and instead of the doctor or nurse coming out to see the patient, they can explain how the patient is, what condition the patient is, or what the problem is and they can actually give them advice through that. Rather than coming out. If I’m wrong then I apologise (carer).

## Discussion

This study developed and tested theories related to the uptake and sustainability of telemedicine in care homes that coincided with Weiner’s (2009) theory of organisational readiness for change. The main contextual factors that facilitated change efficacy and commitment were ‘inner setting’ factors [46]; the most pertinent being leadership, social links within the home and psychological safety as these promoted shared learning and confidence in using the technology.

### Change commitment

This research identified that the uptake and sustainability of videoconferencing is increased where there is a common culture of understanding, trust, and purpose, and where staff are committed to the goals of the care home [40]. With the importance of having a common sense of purpose to increase the uptake and sustainability of interventions amongst care staff having previously been noted [47].

This project found that the value of videoconferencing to the care home is generated through a perceived advantage of the service relative to other services. The main factors that affected this sense of relative advantage included the quality of the healthcare that was already being received by the resident, and whether or not videoconferencing would improve the speed of access to healthcare. With access to additional expertise, in comparison to the skills of the staff in the care home, being associated with use. The effect of this skills differential has also been identified in other studies such as Gage (2012), who found that homes without on-site nursing staff were much more likely to access health services than those with on-site nursing [48].

Additionally, access to out-of-hours services proved influential when deciding whether or not to use videoconferencing: uptake of videoconferencing was more likely where staff were dissatisfied with out-of-hours services. This supports existing research, which highlights the importance of developing an intervention that is in line with care priorities to increase uptake and sustainability [49]. For example, Goodman (2016) found introducing interventions that support the priorities of the care home ensures they are enacted [47]. The uptake and sustainability of interventions is supported by the alignment of priorities, which supports the tension for change [50].

Leadership engagement and champions were invaluable in driving commitment, with the importance of key findings such as; empowerment, communication and leadership qualities being echoed in a recent review on effective leadership in social care [49]. Finally, champions were found to be essential in providing the necessary access to the continuity of support and expertise required for uptake and sustainability of videoconferencing to be achieved [47, 49].

#### Change efficacy

The care home’s perceived ability to implement the service was the second factor found to increase the uptake and sustainability of videoconferencing [39]. This was generated through the availability of resources e.g. staff. This is supported by Stern et al. (2014), who found high staff turnover impeded the uptake of a telemedicine intervention for pressure ulcers [51]. Greenhalgh et al. (2017) also highlighted that the ability to innovate is affected by organisational capacity [50]. Clarity of leadership and effective team-working have also been shown to facilitate organisational innovation [52].

Additionally the care homes' ability to effect change was also influenced by the ratio of senior staff available, with previous research highlighting the importance of having available leadership and staff with appropriate skill sets [20, 50, 53].

Findings from this research suggest a positive communication culture increases trust and encourages a supportive learning climate. This is in keeping with previous research that found that increased communication (informal and formal) between staff, improved resident outcomes [20, 49]. Informal training was supported by low staff turnover and increased team stability, as the stronger social links in the home made staff feel better able to learn how to use the system without fear of criticism. Edmondson et al. (2001) supports this finding, suggesting that increased psychological safety increases with greater team stability. This was seen as necessary as risk-taking is required when implementing a new intervention and when undertaking the necessary training [54].

#### Contextual factors

Three main contextual factors supporting the care homes' readiness for change were identified through this research [40], these were; encouraging a positive communication culture in the care home to help foster trust [49], to increase psychological safety, and encourage a care home culture that fosters shared learning [49, 54, 55]. This has been shown to be achievable through authentic leadership [44, 49]. This finding is supported by Anderson (2003) who found increased communication in management practices improved resident outcomes [56].

Linked to this, recruiting staff who are interested in the long-term goals of the home [57] and who have a higher level of organisational commitment may increase team stability [42, 54]. Recruiting before the home is short-staffed was suggested as being ideal as this allowed the manager to take their time over the selection process. This is supported by research suggesting that small independent providers may be more likely to recruit through informal channels, resulting in more staff being recruited who may be a more suitable fit for the organisation [57] and might have existing strong social bonds with members of staff. This should be explored further as it may be a contributing factor that was not recognised.

Finally, a need for greater access to information about videoconferencing was identified; to enable managers to make more informed decisions [58].

The findings suggest that, overall, videoconferencing is a viable tool as long as certain prerequisites are in place; for example, champions to drive implementation and commitment from managers and staff.

## Limitations

The findings should be treated as indicative, as it is possible that there are additional insights that might be gained by applying the same methodology to a larger data set. The study explored a large number of partial candidate theories, only a few of which were able to be explored in great depth [59]. Where the evidence is weaker, these theories require further refinement and testing [59]. This study might have benefitted from a larger number of additional factors being explored in more depth. For example, a remote service provider perspective was not included.

## Conclusions and recommendations

The uptake and sustainability of videoconferencing in care homes was found to be facilitated by whether or not the care home staff value videoconferencing, and whether they have the resources as a team to implement it [20, 40, 50]. Care home culture, communication within the care home, the knowledge that staff have of videoconferencing and their prior experiences of accessing external services were found to be pertinent in establishing the care homes perceived readiness for change. Effective staff recruitment was also highlighted as being pertinent, but requires further exploration [20, 50]. The findings could usefully be applied in the context of implementing videoconferencing in care homes to help improve successful implementation and sustainability, and some key principles might translate to other settings.

## Data Availability

The datasets generated and analysed during the current study are not publicly available to ensure participant confidentiality but are available from the corresponding author on reasonable request.

## Abbreviations

CMOCs: Context mechanism outcome configurations
NHS: National Health Service
CFIR: Consolidated framework for implementation research
MRT: Middle-range theory

## References

[1] Care Quality Commission. The state of adult social care services 2014 to 2017: Findings from CQC’s initial programme of comprehensive inspections in adult social care2017 28.9.2017 [cited 2017 28.9.2017]. Available from: http://www.cqc.org.uk/sites/default/files/20170703_ASC_end_of_programme_FINAL2.pdf.

[2] Smith P, Sherlaw-Johnson C, Ariti C, Bardsley M. Focus on: Hospital admissions from care homes London: The Health Foundation and the Nuffield Trust.; 2015. Available from: http://www.health.org.uk/sites/default/files/QualityWatch_FocusOnHospitalAdmissionsFromCareHomes.pdf.

[3] Unison. The Damage: Care in Crisis2016 [cited 2017 10.10.2017]. Available from: https://www.unison.org.uk/content/uploads/2016/11/24149_The_Damage_care_in_crisis_web.pdf.

[4] Hex N, Wright D (2015). Evaluation of telehealth interventions for care homes in Airedale.

[5] NHS England. Five Year Forward View2014 23/12/2015. Available from: https://www.england.nhs.uk/wp-content/uploads/2014/10/5yfv-web.pdf.

[6] NHS England. The NHS Long Term Plan2019 01.02.20. Available from: https://www.longtermplan.nhs.uk/wp-content/uploads/2019/08/nhs-long-term-plan-version-1.2.pdf.

[7] Johnston D, Jones BN (2001). Telepsychiatry consultations to a rural nursing facility: a 2-year experience. J Geriatr Psychiatry Neurol.

[8] Coelho JJ, Arnold A, Nayler J, Tischkowitz M, MacKay J. An assessment of the efficacy of cancer genetic counselling using real-time videoconferencing technology (telemedicine) compared to face-to-face consultations. European journal of cancer (Oxford, England : 1990). 2005;41(15):2257–61. 10.1016/j.ejca.2005.06.020 16176873

[9] Cruickshank J, Paxman J. Yorkshire and the Humber Telehealth Hub: Project Evaluation. January 2013.: 2020Health.org; 2013.

[10] NHS England. New care models – vanguard sites: NHS England; 2015. Available from: https://www.england.nhs.uk/ourwork/futurenhs/new-care-models/.

[11] NHS England. The framework for enhanced health in care homes; 2016. Available from: https://www.england.nhs.uk/wp-content/uploads/2016/09/ehch-framework-v2.pdf.

[12] McGibbon F, Dorrian C, O'Keeffe R (2013). Lochaber telemedicine clinic: a new approach managing dementia in care homes. Int J Integr Care..

[13] Low JA, Beins G, Lee KK, Koh E (2013). Last moments of life: Can telemedicine play a role?. Palliat Support Care.

[14] Department of Health. End of Life Care Strategy: Promoting high quality care for all adults at the end of life 2008. Available from: https://www.gov.uk/government/uploads/system/uploads/attachment_data/file/136431/End_of_life_strategy.pdf.

[15] Garnett P, Hanson G. Post Implementation Review: Telemedicine for Care Homes Project. Unpublished; 2016.

[16] Flodgren G, Rachas A, Farmer A, Inzitari M, Shepperd S. Interactive telemedicine: effects on professional practice and healthcare outcomes|Cochrane. Cochrane Database of Systematic Reviews. 2015(9). 10.1002/14651858.CD002098.pub2 PMC6473731 26343551

[17] Czaja SJ (2016). Long-term care services and support systems for older adults: the role of technology. Am Psychol.

[18] Newbould L, Mountain G, Hawley MS, Ariss S (2017). Videoconferencing for health care provision for older adults in care homes: a review of the research evidence. Int J Telemed Appl.

[19] Hall A, Wilson C, Stanmore E, Todd C (2017). Implementing monitoring technologies in care homes for people with dementia: a qualitative exploration using normalization process theory. Int J Nurs Stud.

[20] Goodman C, Sharpe R, Russell C, Meyer J, Gordon A, Dening T, et al. Care home readiness: a rapid review and consensus workshops on how organisational context affects care home engagement with health care innovation. Online: University of Hertfordshire; 2017 29.05.2017.

[21] Pawson R. The science of evaluation: a realist manifesto. SAGE Publications Ltd, London; 2013.

[22] Manzano A (2016). The craft of interviewing in realist evaluation. Evaluation.

[23] Foley G, Timonen V (2015). Using grounded theory method to capture and analyze health care experiences. Health Serv Res.

[24] Fletcher A (2017). Applying critical realism in qualitative research: methodology meets method. Int J Soc Res methodol.

[25] Pawson R, Tilley N (1997). Realistic evaluation.

[26] Patton M (2011). Developmental evaluation: applying conplexity concepts to enhance innovation and use.

[27] Care Quality Commission. Types of care home: Care Quality Commission; 2015. Available from: http://www.cqc.org.uk/content/care-homes.

[28] Binks R. Fw: Airedale telemedicine vanguard - evaluation - lknewbould1@sheffield.ac.uk - University of Sheffield Mail. In: Newbould L, editor. E-mail with most recent figures on videoconferencing use. ed2017. p. 1.

[29] Campbell S. Comparative case study. Encyclopedia of Case Study Research. Online: SAGE Publications, Inc.; 2012. p. 175–6.

[30] Goodrick D. Comparative Case Studies2014 17.7.2017. Available from: http://devinfolive.info/impact_evaluation/ie/img/downloads/Comparative_Case_Studies_ENG.pdf.

[31] Rycroft-Malone J, McCormack B, Hutchinson AM, DeCorby K, Bucknall T, Kent B (2012). Realist synthesis: illustrating the method for implementation research. Implem Sci.

[32] Newbould L, Mountain G, Ariss S, Hawley SM (2019). Remote health care provision in care homes in england: an exploratory mixed methods study of Yorkshire and the Humber. Technologies.

[33] Proctor E, Silmerem H, Raghavan R, Hovmand P, Aarons G, Bunger A (2011). Outcomes for implementation research: conceptual distinctions, measurement challenges, and research Agenda. Adm Policy Ment Health.

[34] CFIR Research Team. Consolidated Framework for Implementation Research (CFIR): Constructs Ann Arbor, United States: The Consolidated Framework for Implementation Research; 2020 [updated 2016-10-25. Available from: https://cfirguide.org/constructs/.

[35] Braun V, Clarke V (2008). Using thematic analysis in psychology. Qual Res Psychol.

[36] Dalkin SM, Greenhalgh J, Jones D (2015). What’s in a mechanism? Development of a key concept in realist evaluation. Implementation Sci.

[37] Wong G, Westhorp G, Manzano A, Greenhalgh J, Jagosh J, Greenhalgh T (2016). RAMESES II reporting standards for realist evaluations. BMC Med.

[38] The RAMESES Projects. Standards and Training Materials Online: RAMSES; 2013–2017. Available from: https://www.ramesesproject.org/Standards_and_Training_materials.php.

[39] Salter KL, Kothari A (2014). Using realist evaluation to open the black box of knowledge translation: a state-of-the-art review. Implem Sci..

[40] Weiner B (2009). A theory of organizational readiness for change. Implem Sci.

[41] Marylène G, Molson J, Deci EL (2005). Self-determination theory and work motivation. J Organ Behav.

[42] Laschinger H, Finegan J, Shamian J (2001). The impact of workplace empowerment, organizational trust on staff nurses' work satisfaction and organizational commitment. Health Care Manage Rev.

[43] Kanter R (1987). Men and women of the corporation revisited. Manag Rev.

[44] Avolio B, Gardner W, Walumbwa F, Luthans F, May D (2004). Unlocking the mask: a look at the process by which authentic leaders impact follower attitudes and behaviors - ScienceDirect. Leadership Quart.

[45] Schonfeld P, Preusser F, Margraf J (2017). Costs and benefits of self-efficacy: Differences of the stress response and clinical implications. Neurosci Biobehav Rev.

[46] MacKinnon DP (2011). Integrating mediators and moderators in research design. Res Soc Work Pract.

[47] Goodman C, Dening T, Gordon AL, Davies SL, Meyer J, Martin FC (2016). Effective health care for older people living and dying in care homes: a realist review. BMC Health Serv Res..

[48] Gage H, Dickinson A, Victor C, Williams P, Cheynel J, Davies S (2012). Integrated working between residential care homes and primary care: a survey of care homes in England. BMC Geriatr.

[49] Smith T, Fowler-Davis S, Nancarrow S, Ariss S, Enderby P (2018). Leadership in interprofessional health and social care teams: a literature review. Leadersh Health Serv.

[50] Greenhalgh T, Wherton J, Papoutsi C, Lynch J, Hughes G, A'Court C (2017). Beyond adoption: a new framework for theorizing and evaluating nonadoption, abandonment, and challenges to the scale-up, spread, and sustainability of health and care technologies. J Med Internet Res.

[51] Stern A, Mitsakakis N, Paulden M, Alibhai S, Wong J, Tomlinson G (2014). Pressure ulcer multidisciplinary teams via telemedicine: a pragmatic cluster randomized stepped wedge trial in long term care. BMC Health Serv Res..

[52] West M, Borrill C, Dawson J, Brodeck F, Shapiro D, Haward B (2003). Leadership clarity and team innovation in health care. Leadersh Quart.

[53] Davison T, Karantzas G, Mellor D, McCabe M, Mrkic D (2013). Staff-focused interventions to increase referrals for depression in aged care facilities: a cluster randomized controlled trial. Aging Mental Health.

[54] Edmondson AC, Bohmer RM, Pisano GP (2001). Disrupted routines: team learning and new technology implementation in Hospitals. Admin Sci Quart.

[55] Zeffane R, Tipu S, Ryan J. Communication, Commitment & Trust: Exploring the Triad. 6. 2011.

[56] Anderson RA, Issel LM, McDaniel RR (2003). Nursing homes as complex adaptive systems: relationship between management practice and resident outcomes. Nurs Res.

[57] Rubery J, Hebson G, Grimshaw D, Carroll M, Smith L, Marchington L, et al. The recruitment and retention of a care workforce for older people - Social Care Online Manchester: Manchester University Business School; 2011. Available from: https://www.kcl.ac.uk/sspp/policy-institute/scwru/dhinitiative/projects/ruberyetal2011recruitmentfinal.pdf.

[58] Maczka M, Parry D, Curry R, Ansett C. Technology in Care Homes: The SEHTA Review. Online: SEHTA; 2016 30.09.2016.

[59] Flynn R, Schick-Makaroff K, Levay A, Greenhalgh J (2020). Developing an initial program theory to explain how patient-reported outcomes are used in health care settings: methodological process and lessons learned. Int J Qualit Methods..

